# Expression of *Calca* gene-derived peptides in the murine taste system

**DOI:** 10.64898/2026.01.16.700005

**Published:** 2026-01-20

**Authors:** Salin Raj Palayyan, Abdul Hamid Siddiqui, Sunil Kumar Sukumaran

**Affiliations:** 1 Department of Nutrition and Health Sciences, University of Nebraska-Lincoln, Lincon, NE, 68583

**Keywords:** Neuropeptide, Procalcitonin, CGRP, Gustation

## Abstract

The Calcitonin Related Polypeptide Alpha (*Calca*) gene is a source of four biologically active peptides with varied physiological roles. Alternative splicing of the *Calca* messenger RNA generates either prepro calcitonin gene related peptide (CGRP) or preprocalcitonin encoding transcripts. Proteolytic processing of preprocalcitonin generates procalcitonin, calcitonin and katacalcin. Calcitonin is a ligand for the G-protein coupled receptor calcitonin receptor (CALCR) while CGRP is a ligand for the CGRP receptor (CGRP1R) formed by the calcitonin receptor like receptor (CALCRL)receptor activity modifying protein 1 (RAMP1) complex. Interestingly, procalcitonin too, is a ligand for the CGRP1R where it can antagonize CGRP. CGRP expression in taste neurons has been documented and is posited to regulate taste signaling. Single cell and bulk RNASeq of taste papillae revealed that the preprocalcitonin but not the CGRP transcript is expressed in *Tas1r3*- expressing type II taste cells, while *Calcrl* (but not *Calcr*) and *Ramp1* are expressed in stem/progenitor and type I cells in the circumvallate papillae. The CGRP1R is also expressed by fibroblasts in the lingual mesenchyme. We confirmed this expression pattern using quantitative polymerase chain reaction (qPCR), RNAScope and immunohistochemistry. qPCR of geniculate and nodose-petrosal ganglia revealed that both express *Cgrp* and CGRP1R subunit mRNAs, but not procalcitonin and *Calcr*. This interesting expression patterns suggests that procalcitonin and CGRP might reciprocally regulate the CGRP1R in taste cells and lingual fibroblasts and thereby influence taste signaling, taste cell regeneration and the taste microbiome.

## Introduction

The *Calca* gene is a source of up to four peptide signaling molecules. Tissue specific alternative splicing of *Calca* transcript generates mRNAs coding for either calcitonin gene related peptide (preproCGRP, proteolytically processed to alpha CGRP) or preprocalcitonin (prePCT).^1,2^ PrePCT is proteolytically processed to generate procalcitonin (PCT), which is further processed to generate calcitonin and katacalcin.^3^ All four belong to a larger group of peptides that include beta CGRP, amylin, adrenomedullin and intermedin, which are ligands for receptors formed by the GPCRs calcitonin receptor (CALCR) or calcitonin receptor like receptor (CALCRL).^4^ CALCR and CALCRL combine with one of three receptor activity modifying proteins (RAMP1, RAMP2 or RAMP3) to form receptor complexes specific to a subset of these peptides. For example, calcitonin is a ligand for CALCR (without an associated RAMP subunit), while CGRP is a ligand for both CALCR+ RAMP1 and CALCRL+RAMP1 (this being its primary receptor, henceforth designated CGRP1R).^4,5^ CGRP is an exceptionally well-studied neuropeptide; it is expressed in virtually all peripheral sensory neurons, many central neurons, and some non-neuronal cells.^6–8^ It serves a wide variety of functions including nociception, neurogenic inflammation, immunity, vasodilation, wound healing, regulation of the microbiome etc.^6–8^ It is a potent neuro-immune modulator and was recently shown to modulate antigen sampling by M cells in the Peyer’s patch.^9^ It displays antimicrobial activity against several gram-negative and gram-positive bacteria and Candida albicans, which adds a further dimension to its immunomodulatory and wound healing roles.^10,11^ From a clinical standpoint, its prominent role in triggering migraine has generated significant scientific interest.^6^ This has led to the development of small molecule- and monoclonal antibody- based CGRP1R antagonists to treat migraines.^12,13^ Interestingly, clinical studies have suggested that one-quarter of migraine patients have altered taste perception.^14^ Indeed, CGRP was shown to shape taste transduction by inducing 5-HT secretion by type III taste cells in a phospholipase C-dependent manner.^15^

Calcitonin and katacalcin are key regulators of calcium and phosphate levels in blood and bone.^3,16,17^ PCT is a much less studied neuropeptide. It is not widely secreted in the steady state; it may regulate bone density by suppressing osteoclast (macrophage) migration and maturation.^18^ Interestingly, sepsis-associated cytokine storm is preceded by ubiquitous upregulation of PCT expression, leading to its adoption as an early sepsis marker.^19–22^ PCT is a partial agonist of CGRP1R and exerts its mediator role in sepsis through this receptor. It also partially antagonizes CGRP at this receptor.^23–25^ However, its biological role(s) in both the steady state and in sepsis remains enigmatic. Single cell RNASeq (scRNASeq) and bulk RNASeq of the circumvallate papillae (CVP) done in our lab showed that the prePCT encoding *Calca*-transcript is highly expressed in type II taste cells, while subunits of the CGRP1R are expressed in taste stem cells, type I taste cells and lingual mesenchymal fibroblasts. The CGRP transcript and *Calcr* were not expressed in taste tissues. This was confirmed using qPCR, RNAScope and immunohistochemistry. On the other hand, the nodose- petrosal and geniculate ganglia that innervate taste buds were shown to express the CGRP- transcript and CGRP1R subunits, but not the prePCT transcript and *Calcr*. This intriguing expression pattern suggests that taste cell derived PCT and taste nerve derived CGRP may reciprocally modulate CGRP1R in the taste papillae.

## Methods

### Animals.

8–10 weeks old C57BL/6J mice (The Jackson Laboratory, Bar Harbor, ME) were used for this study. Animals were housed in a specific pathogen free vivarium with a 12-h light/dark cycle and open access to food and water. All animal experiments were performed in accordance with the National Institutes of Health guidelines for the care and use of animals in research and reviewed and approved by the Institutional Animal Care and Use Committee at University of Nebraska-Lincoln (protocols: 2610 and 2366).

### Bulk RNASeq of taste papillae.

Pooled taste buds from CVP and fungiform papillae (FFP) of C57BL/6 mice (n=3 each) were excised from CVP sections using laser micro dissection, and full length bulk RNASeq libraries were prepared using the Ovation RNASeq system V2 (Tecan biosystems, Morgan Hill, CA) per manufacturer instructions. Indexed illumina sequencing libraries were prepared and sequenced in a Hiseq 2000 sequencer (Illumina Inc, San Diego, CA). Raw sequences were aligned to the mouse reference genome (version GRCm38.p3) using the STAR program with default settings and Gencode M24.gtf as the splice junction annotation file, and the reads mapping to genes were counted using the featureCounts package.^26,27^ Sashimi plots to show alignment of reads were generated using the integrative genome viewer.^28^

### scRNASeq of taste papillae.

Chromium^™^ Single Cell 3′ Solution (10x Genomics Inc, Pleasanton, CA, Cat. no. PN-1000268) was used for scRNASeq analysis.^29^ Single cell preparation from CVP was done as previously described.^30,31^ A protease cocktail was injected under the lingual epithelium of excised tongue (n=16 mice) and incubated at 37°C for ten minutes. The epithelia were peeled, the CVP were excised and minced to form single cells. Single cell capture, library preparation, sequencing, and primary analyses of sequencing data were done using 10X genomics protocols, and secondary analysis was done using the Seurat package in R.^32^

### RNAScope Hiplex assay.

RNAscope assay was done using the Hiplex fluorescent assay kit for mice (Advanced Cell Diagnostics, Hayward, CA, Cat. no. 324443) with indicated probes (Table S1) as previously described using the manufacturer’s instructions.^33^ Positive and negative control probes were run in parallel to test probes to ensure proper hybridization and imaging conditions were attained in our experiments. Confocal images were captured using a Nikon A1R-Ti2 confocal laser scanning microscope using NIS-Elements A1R software image acquisition and analysis software, using 40/60X objectives. Images were taken using a sequential channel series setting to minimize cross-channel signal, and the channels used were: GFP 488, TxRed 550, Cy5 650 nm. Z-series stack with 10 images per stack was captured at a step size of 1 μm. Acquisition parameters [i.e., gain, offset, photomultiplier tube (PMT) setting] were held constant for experiments. Colocalization counts were made using QuPath software.^34^ Cell boundaries were detected automatically based on DAPI staining. The fidelity of each cell boundary was confirmed by manual inspection. The number of fluorescent spots in each channel (that corresponds to individual mRNA molecules) per cell were extracted and used for colocalization counting. Data from more than two non-consecutive sections from two mice were pooled.

### Immunohistochemistry

Standard immunohistochemical techniques were used as previously described.^33,35^ Briefly, frozen sections were rehydrated with PBS. Nonspecific binding was blocked with SuperBlock Blocking Buffer (Thermo scientific, Waltham, MA, Cat. no. 37580) at room temperature for 1 h. Sections were incubated with primary antibodies overnight at 4 °C in a humidified chamber. After three 15-min washes with PBST, slides were incubated for 1 h at room temperature with one of the following fluorescent secondary antibodies in blocking buffer. All double-immunofluorescent labeling was done with combinations of the secondary antibodies along with DAPI (1:1,000; Invitrogen^™^, Thermo scientific, Waltham, MA, Cat. no. D1306) to label cell nuclei for cell counting. The primary and secondary antibodies and their concentrations used in this study are listed in Table S2.

Double labeling with two antibodies made from the rabbit (LRMP+PCT and T1R3+PCT), was done as described before.^36^ After blocking, the sections were incubated in succession: first primary anti-rabbit antibody overnight at 4 °C, first fluorescence Fab fragment secondary for 1 h at room temperature, unlabeled anti-rabbit antisera [AffiniPure Fab fragment donkey anti-rabbit IgG (H+L)] for 3 h at room temperature to ensure that all binding sites in the first primary antibody are occupied, second primary anti-rabbit antibody overnight at 4 °C, and then second fluorescence Fab fragment secondary for 1 h at room temperature. Controls for the double immunohistochemistry experiments with two rabbit primary antibodies were done without donkey Fab fragment incubation following the first secondary antibody and omitting the second primary antibody (Figure S6 I & II, control A) or with donkey Fab fragment incubation and omitting the second primary antibody (Figure S6 I & II, Control B) to show adequate blocking of rabbit IgG by donkey Fab fragment. Confocal imaging was done as described above for RNAScope. Colocalization for each taste cell marker was done using images from at least 2 sections showing entire CVP and FOP (n=1 mouse). Only those taste cells for which the entire cell body and nucleus could be visualized were counted.

## Western Blot

CVP tissue from three animals was isolated and homogenized in RIPA lysis and extraction buffer (Thermo scientific, Waltham, MA, Cat. no. 89900). Protein quantification was done using BCA method (Thermo scientific, Waltham, MA, Cat. no. 23227). Lysate were then mixed with 4 X laemmli buffer (Bio-Rad Laboratories Inc, Hercules, CA, Cat. no.1610747), and 30 μg of protein run on SDS-PAGE, followed by transfer onto nitrocellulose membrane. Blots were blocked in Blocker (Thermo scientific, Waltham, MA, Cat no. 37520) and incubated with PCT primary antibody (Table S2) at 4 °C overnight. Incubation with secondary antibody was done for 1 h and imaged on Odyssey F Imager (LI-COR Inc. Lincoln, NE).

### PCR and qPCR.

Total RNA was isolated from freshly dissected geniculate and nodose-petrosal ganglia, taste papillae or NT epithelium using the Quick-RNA Microprep kit (Zymo Research Corp, Irvine, CA, Cat. no. R1050) with on-column DNA digestion. cDNAs were prepared from total RNAs using SuperScript IV VILO Master Mix kit (Thermo Fisher, Waltham, MA, Cat. no. 11756050). End point PCR and qPCR were done as previously described. Exon-exon junction spanning primers were designed whenever possible to avoid amplification from any contaminating genomic DNA. A minimum of three biological replicates were used for all cDNA samples. The ratio of the log10 of the average δ-cycle threshold (Ct) value (difference between Ct values of *Bact* and each gene of interest was plotted. Primers used are shown in Table S3.

## Results

### Bulk RNASeq and PCR results to show the expression of Calca transcripts and receptors in taste papillae and ganglia.

**Figure 1.**
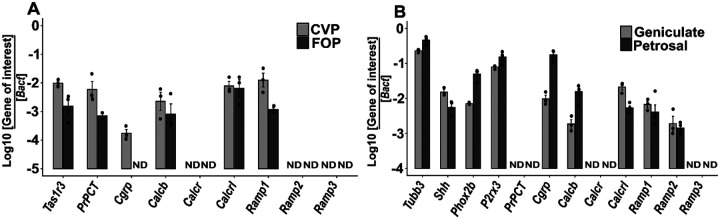
qPCR profiling of *Calca and Calcb* transcripts and their receptors in cDNA from taste papillae and sensory ganglia. A) Strong expression of *PrPCT, Calcb*, *Calcrl* and *Ramp1* is observed in both CVP and FOP. Weak expression of *Cgrp* is observed in CVP, while it is undetectable in FOP. *Calcrl and Ramp1* are expressed in both CVP and FOP while *Calcr, Ramp2 and Ramp3 are* not detected in either CVP or FOP. *Tas1r3* is used as a control to demonstrate the quality of taste cDNA. B) qPCR of above transcripts in geniculate and nodose-petrosal ganglia. *Cgrp* and *Calcb* are expressed in both ganglia, with stronger expression observed in the nodose-petrosal ganglion. *Calcrl, Ramp1 and Ramp2* are expressed in both ganglia and similar levels. Expression of *Ramp3*, *PrPCT* and *Calcr* is not observed in either ganglion. The taste ganglion marker genes *Tubb3, Shh, Phox2b*, and *P2rx3* are used to demonstrate the quality of ganglia cDNA. The expression of each gene is plotted as the logarithm of the ratio between its cycle threshold value and that of *Bact*. ND= not detected.

Analysis of bulk RNASeq data from CVP taste buds isolated by laser microdissection showed that the *Calca* transcript is strongly expressed in CVP. Alignment of the RNASeq reads to the *Calca* genomic locus showed that no reads mapped to the preproCGRP specific exon 5, while large number of reads aligned to prePCT specific exon 4 (Figure S1). This observation was confirmed using end point PCR using primers for the *Cgrp* and prePCT transcripts (Figure S2A), which showed amplification for the prePCT transcript in CVP and foliate papillae (FOP), but not the FFP. *Cgrp* transcript is expressed at very low levels or were undetectable in all three taste papillae. We could readily detect transcripts for *Calcrl* and *Ramp1* in all three taste papillae and for *Ramp2* in CVP, but not for *Calcr* and *Ramp3*. The transcript for *Calcb* that encodes beta CGRP is expressed in all three taste papillae. None of the transcripts were expressed in non-taste lingual epithelium (NT, Figure S2A). Next, we checked the expression of these transcripts in the geniculate and nodose- petrosal ganglia that innervate the FFP and CVP and FOP respectively. The prePCT transcript and *Calcr* are not expressed in either of them, while the *Cgrp* transcript, *Calcrl, Calcb*, *Ramp1*, and *Ramp2* were detected in both (Figure S2B). Next, we quantified the expression of these transcripts in all four tissues using qPCR. Consistent with the end point PCR results, robust expression of prPCT is detected in both papillae and weak expression of *Cgrp* is detected in the CVP but not FOP (Figure 1A). Conversely, *Cgrp*, but not prPCT transcript is strongly expressed in both taste ganglia (Figure 1B). Robust expression of *Calcrl, Calcb and Ramp1* were detected in both the taste papillae and ganglia, while *Calcr and Ramp3* are not expressed in any sample. Weak *Ramp2* expression is detected in the two ganglia, while it is not expressed in the taste papillae (Figure 1).

### scRNASeq of CVP identifies Calca and CGRP1R subunit expressing taste and mesenchymal cells.

To identify taste cells that express *Calca* and CGRP1R subunits, we turned to scRNAseq data from CVP. We found that *Calca* is expressed only in mature and immature sweet taste cells. *Calcrl* is expressed in subtypes of type I cells and taste stem cells. *Ramp1* expression was low overall, while the regulatory subunit of the CGRP1R, *Crcp* is expressed widely across all cell types (Figure S3).

### Western blot and histological analyses confirm the expression of Calca and Calcrl in taste cells.

**Figure 2.**
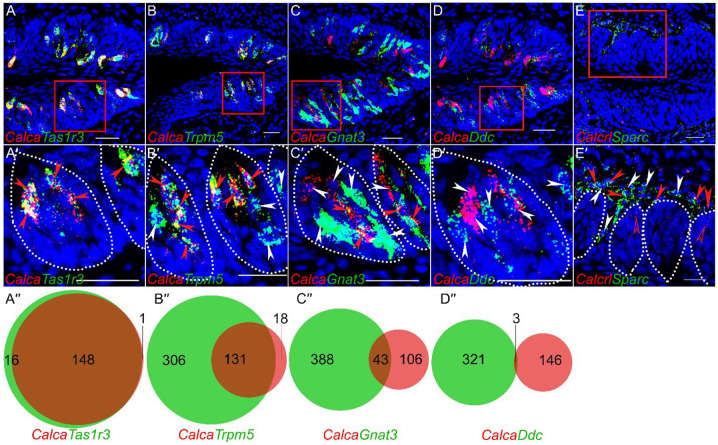
RNAScope analysis of *Calca* and *Calcrl* gene expression in CVP. RNAscope Hiplex fluorescence assay was used to determine the coexpression of *Calca* with the taste cell markers *Tas1r3* (A-A′), *Trpm5* (B-B′)*, Gnat3* (C-C′) and *Ddc* (D-D′). The areas highlighted in red boxes in the top row is magnified in the bottom rows for each set. Taste buds are highlighted by white dotted lines. A″-D″ are venn diagrams showing the number of taste cells that co express or singly express the indicated marker genes and *Calca*. Data are from two non-consecutive sections from two mice. E & E′ shows the expression of *Calcrl* in fibroblasts adjacent to basal taste cells marked by *Sparc*. Strong coexpression of *Calca* is observed with *Tas1r3* and *Trpm5*, less strong coexpression is observed with *Gnat3* and negligible coexpression is observed with *Ddc*. *Calcrl* expression is observed in basal (presumably stem/progenitor) cells in the taste buds and adjacent fibroblasts. Filled white arrowheads highlights single positive cells, filled red arrowheads highlights double positive cells, and open red arrowheads depicts basal cells in taste buds expressing *Calcrl*. Scale bars = 30 μm.

**Figure 3.**
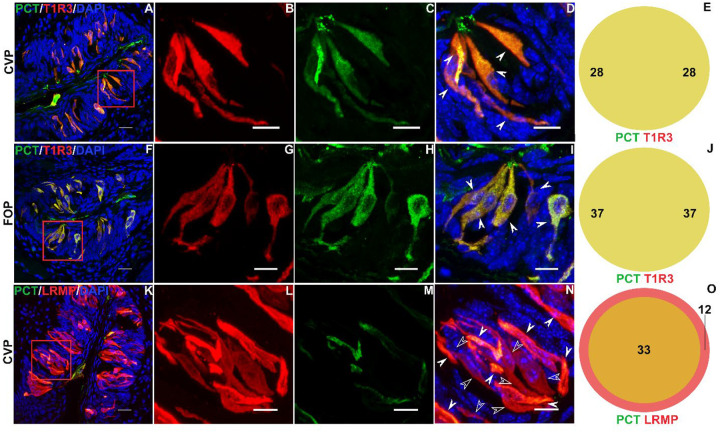
PCT is expressed in type II taste cells. Double labelled immunofluorescence confocal microscopy of CVP and FOP sections with antibodies against PCT (green) and type II taste cell markers T1R3 (red; A–D, F-I) or LRMP (red; K–N). Nuclei are counterstained with DAPI (blue). A, F, K are lower magnification images and dashed red boxes indicate regions shown at higher magnification in panels in the right. (D, H, L) Higher-magnification views of the boxed region showing double positive cells with solid arrows and single positive cells hollow arrows. Colocalization counts are shown in Venn diagrams (E,J,O). Scale bars = 20 μm.

Using western blot analysis using an antibody specific to PCT, we detected its expression in CVP (Figure S4). Next, we turned to histological analysis to confirm the cell type specific patterns of *Calca* and *Calcrl*. RNAscope Hiplex analysis of CVP confirmed the scRNASeq results: *Calca* is strongly coexpressed with the sweet taste receptor subunit *Tas1r3*, with 148/164 (90%) of *Tas1r3* expressing cells coexpressing *Calca*, and 148/149 (99%) *Calca* expressing cells coexpressing *Tas1r3* (Figure 2A– A″)*. Calca* expressing cells also coexpressed the pan type II taste cell marker *Trpm5*, with 131/437 (30%) of *Trpm5* expressing cell coexpressing *Calca*, while 131/149 (88%) of *Calca* expressing cells coexpress *Trpm5* (Figure 2 B– B″). Most type II cells that do not express *Calca* appear to be bitter taste receptor cells, as less than one tenth of *Gnat3* (primarily bitter taste cell marker in CVP) expressing cells expressed *Calca* (Figure 2C– C″). *Calca* is not expressed in type III taste cells marked by *Ddc*. (Figure 2D– D″). *Calcrl* staining is observed in basal (presumably stem/progenitor) cells in the CVP taste buds (Figure 2E, E′). Significant staining for *Calcrl* is also observed in mesenchymal cells close to basal cells of taste buds that stained strongly for the fibroblast marker *Sparc* (Figure 2E, E′). Comparable results were obtained using double labeled immunohistochemistry. Using a PCT specific antibody, we saw strong co expression of PCT with the sweet taste receptor subunit T1R3 (100% in both directions) in CVP (Figure 3 A–E) and FOP (Figure 3 F–J). In addition, PCT is strongly coexpressed with the pan-type II marker LRMP, with all PCT expressing cells expressing LRMP and 33/45 (73%) of LRMP expressing cells coexpressing PCT in the CVP (Figure 3 K–O). In case of LRMP, colocalization is observed primarily with cells that stained strongly with LRMP antibody. The weaker LRMP positive cells primarily expressed GNAT3, which, as alluded to before, is primarily expressed in bitter taste receptor cells in the CVP (Figure S5).

## Discussion

The *Calca* derived peptides CGRP, calcitonin and procalcitonin play key roles in health and disease.^3,6–8,19,21,37,38^ Using a slew of techniques probing the expression of the corresponding mRNAs and proteins, we show that the prePCT encoding transcript is strongly expressed in CVP and FOP, almost exclusively in the *Tas1r3* expressing type II (primarily sweet and umami receptor expressing) taste cells. Curiously, *Calca* expression (both prePCT and *Cgrp* mRNAs) is not observed in FFP (Figure S2). The related *Calcb* gene is expressed in all three taste papillae. All three taste papillae express the CGRP1R subunits *Calcrl* and *Ramp1* (Figure S2, Figure 1). Interestingly, the taste ganglia that innervate the papillae expresses the *Cgrp* but not the prePCT transcript and expresses both the CGRP1R subunits (Figure S2, Figure 1). A small amount of *Cgrp* transcript is detected in the CVP using endpoint PCR and qPCR, although it is likely derived from the nerve endings that innervate the taste papillae rather than taste cells themselves (Figure S2, Figure 1). This is supported by the absence of *Cgrp* transcript in the bulk RNASeq data from CVP, derived from laser microdissected taste buds devoid of contamination from nerve bundles in the CVP core that would be present in the CVP samples used for PCR experiments (Figure S1). CGRP expression in trigeminal neurons is well studied, and it is very likely that trigeminal neurons that innervate the taste papillae also express CGRP, although this has not been experimentally demonstrated to our knowledge.^7^ Thus, it is possible that the bulk of alpha CGRP in the taste papillae is nerve derived. This is also supported by immunolocalization studies of CGRP in rodent, pig and human taste papillae, all of which show CGRP expression restricted to nerve bundles and their termini in and around taste buds.^15,39–41^ PrePCT is processed to generate PCT and then to calcitonin and katacalcin. We were able to detect the robust expression of prePCT transcript using end point and qPCR and could detect PCT protein expression using western blot (Figures S2, S4 and 1). Although the RNAScope probe for *Calca* does not distinguish between the two alternatively spliced mRNAs, we obtained strong staining in TAS1R3- and LRMP- expressing taste cells with the PCT antibody (Figure 2, Figure 3). However, these experiments do not allow us to determine if calcitonin and katacalcin are produced by taste cells. Since both peptides are part of PCT, they cannot be readily distinguished using antibody staining. Notably, their biological roles are distinct from that of PCT; both are well known hormones produced by the parathyroid gland that bind to the calcitonin receptor to regulate bone and serum calcium and phosphorus levels.^16,17^ PCT on the other hand, is an early marker of sepsis, which is only expressed at very low levels in healthy individuals.^19,24^ Notably, we did not detect the calcitonin receptor mRNA *Calcr*, in the taste papillae or the geniculate and nodose-petrosal ganglia (Figure S2, Figure 1). Thus, the available evidence indicates that calcitonin and katacalcin are either not produced by taste cells and nerves or will not be biologically effective in case they are produced. PCT on the other hand, can stimulate CGRP1R, and it also antagonizes CGRP at this receptor.^23–25^ Considering abundant CGRP1R receptor expression in taste papillae and ganglia, PCT is the only peptide generated from taste cell expressed prePCT transcript capable of exerting its biological role.

What are the likely roles of PCT and CGRP in the taste system? The amount of peptides produced by the taste cells will not be sufficient to elevate their circulating levels, and their effects will be mediated by paracrine signaling within the taste papillae. We know a lot about the biological roles of CGRP. One study that looked at the effects of CGRP in isolated taste buds using calcium imaging and bioassay showed that CGRP may regulate taste signaling by regulating 5-HT signaling by type III cells.^15^ We (and the original study) did not detect CGRP1R expression in type III cells. We detected *Crcp* expression in many taste cell types using scRNASeq, which agrees with their findings (Figure S2). Using scRNASeq, we detected CGRP1R expression in type I cells (Figure S2). Type I cells can regulate taste signaling, which is an alternative explanation for CGRP’s ability to regulate taste signaling.^42^ We found that *Calcrl* is expressed in taste stem cells using scRNASeq and RNAScope (Figure S3, Figure 2). However, *Ramp1 was not detected by scRNASeq* which is likely a false negative due to the limitations of scRNASeq, as we could detect it using qPCR. CGRP regulates stem cell maintenance, and CGRP1R expression in basal cells in the taste buds and adjoining fibroblasts raises the possibility that taste nerve derived CGRP regulates taste stem cells directly, and indirectly by regulating adjoining fibroblasts (Figure 2).^43,44^ CGRP can also regulate immunity at the taste cells through its effects on immune cells, epithelial cells and fibroblasts, and can shape the oral microbiome directly through its microbicidal effects.^8,11,37,45–47^ It is also capable of regulating microfold cells that mediate microbial transcytosis in the Peyer’s patch.^9^ We have shown that type II taste cells might mediate immune surveillance similar to microfold cells, and it is possible that CGRP plays a similar role in taste papillae.^33^ Sepsis-associated cytokine storm is preceded by ubiquitous PCT expression, leading to its adoption as an early sepsis marker. However, very little is known about its role in normal physiology. In the skeletal system, it may regulate bone density by suppressing osteoclast (macrophage) migration and maturation.^18^ It’s expression in CVP and FOP but not FFP might provide some clues to its function in taste cells. Unlike the FFP, the CVP and FOP have deep trenches around which the taste buds are arranged. The trenches are relatively less exposed to salivary flux and may be more hospitable to the oral microbiota, including those responsible for halitosis, and plausibly, pathogenic as well. This raises the possibility that PCT might regulate the taste papillae microbiome in much the same manner as CGRP. In addition, it has the potential to regulate taste signaling and taste cell regeneration by regulating type I and stem/progenitor cell expressed CGRP1R. As stated before, PCT is a partial agonist of the CGRP1R, and it can partially antagonize the effects of CGRP at this receptor. Thus, it is plausible that PCT and CGRP reciprocally shape the biological effects CGRP1R signaling in the taste papillae. Thus, the taste papillae might be a suitable model system to determine the biological roles of PCT and its cross talk with CGRP.

## Supplementary Material

1

## Data Availability

The underlying bulk and scRNASeq data are being uploaded to NCBI’s short read archive and will be available very soon.

## References

[1] Lou H. & Gagel R. F. Alternative RNA processing--its role in regulating expression of calcitonin/calcitonin gene-related peptide. J Endocrinol 156, 401–405, doi:10.1677/joe.0.1560401 (1998). 9582495

[2] Lou H. & Gagel R. F. Mechanism of tissue-specific alternative RNA processing of the calcitonin CGRP gene. Front Horm Res 25, 18–33, doi:10.1159/000061000 (1999). 10941400

[3] Naot D., Musson D. S. & Cornish J. The Activity of Peptides of the Calcitonin Family in Bone. Physiol Rev 99, 781–805, doi:10.1152/physrev.00066.2017 (2019). 30540227

[4] Hay D. L., Garelja M. L., Poyner D. R. & Walker C. S. Update on the pharmacology of calcitonin/CGRP family of peptides: IUPHAR Review 25. Br J Pharmacol 175, 3–17, doi:10.1111/bph.14075 (2018). 29059473 PMC5740251

[5] Pioszak A. A. & Hay D. L. RAMPs as allosteric modulators of the calcitonin and calcitonin-like class B G protein-coupled receptors. Adv Pharmacol 88, 115–141, doi:10.1016/bs.apha.2020.01.001 (2020). 32416865 PMC7374980

[6] Russo A. F. & Hay D. L. CGRP physiology, pharmacology, and therapeutic targets: migraine and beyond. Physiol Rev 103, 1565–1644, doi:10.1152/physrev.00059.2021 (2023). 36454715 PMC9988538

[7] Russell F. A., King R., Smillie S. J., Kodji X. & Brain S. D. Calcitonin gene-related peptide: physiology and pathophysiology. Physiol Rev 94, 1099–1142, doi:10.1152/physrev.00034.2013 (2014). 25287861 PMC4187032

[8] Lu Y. Z., Nayer B., Singh S. K., Alshoubaki Y. K., Yuan E., Park A. J., Maruyama K., Akira S. & Martino M. M. CGRP sensory neurons promote tissue healing via neutrophils and macrophages. Nature 628, 604–611, doi:10.1038/s41586-024-07237-y (2024). 38538784 PMC11023938

[9] Lai N. Y., Musser M. A., Pinho-Ribeiro F. A., Baral P., Jacobson A., Ma P., Potts D. E., Chen Z., Paik D., Soualhi S., Yan Y., Misra A., Goldstein K., Lagomarsino V. N., Nordstrom A., Sivanathan K. N., Wallrapp A., Kuchroo V. K., Nowarski R., Starnbach M. N., Shi H., Surana N. K., An D., Wu C., Huh J. R., Rao M. & Chiu I. M. Gut-Innervating Nociceptor Neurons Regulate Peyer’s Patch Microfold Cells and SFB Levels to Mediate Salmonella Host Defense. Cell 180, 33–49 e22, doi:10.1016/j.cell.2019.11.014 (2020). 31813624 PMC6954329

[10] El Karim I. A., Linden G. J., Orr D. F. & Lundy F. T. Antimicrobial activity of neuropeptides against a range of micro-organisms from skin, oral, respiratory and gastrointestinal tract sites. J Neuroimmunol 200, 11–16, doi:10.1016/j.jneuroim.2008.05.014 (2008). 18603306

[11] N’Diaye A. R., Leclerc C., Kentache T., Hardouin J., Poc C. D., Konto-Ghiorghi Y., Chevalier S., Lesouhaitier O. & Feuilloley M. G. Skin-bacteria communication: Involvement of the neurohormone Calcitonin Gene Related Peptide (CGRP) in the regulation of Staphylococcus epidermidis virulence. Sci Rep 6, 35379, doi:10.1038/srep35379 (2016). 27739485 PMC5064375

[12] Caronna E., Alpuente A., Torres-Ferrus M. & Pozo-Rosich P. CGRP monoclonal antibodies and CGRP receptor antagonists (Gepants) in migraine prevention. Handb Clin Neurol 199, 107–124, doi:10.1016/B978-0-12-823357-3.00024-0 (2024). 38307640

[13] Edvinsson L., Haanes K. A., Warfvinge K. & Krause D. N. CGRP as the target of new migraine therapies - successful translation from bench to clinic. Nat Rev Neurol 14, 338–350, doi:10.1038/s41582-018-0003-1 (2018). 29691490

[14] Kelman L. Osmophobia and taste abnormality in migraineurs: a tertiary care study. Headache 44, 1019–1023, doi:10.1111/j.1526-4610.2004.04197.x (2004). 15546266

[15] Huang A. Y. & Wu S. Y. Calcitonin Gene-Related Peptide Reduces Taste-Evoked ATP Secretion from Mouse Taste Buds. J Neurosci 35, 12714–12724, doi:10.1523/JNEUROSCI.0100-15.2015 (2015). 26377461 PMC6795200

[16] Zdrojewicz Z. & Januszewski A. [Katacalcin--structure, secretion and clinical significance]. Postepy Hig Med Dosw 48, 371–380 (1994). 7638091

[17] Kaneider N. C., Egger P., Wiedermann F. J., Ritter M., Woll E. & Wiedermann C. J. Involvement of cyclic adenosine monophosphate-dependent protein kinase A and pertussis toxin-sensitive G proteins in the migratory response of human CD14+ mononuclear cells to katacalcin. J Bone Miner Res 17, 1872–1882, doi:10.1359/jbmr.2002.17.10.1872 (2002). 12369791

[18] Baranowsky A., Jahn D., Jiang S., Yorgan T., Ludewig P., Appelt J., Albrecht K. K., Otto E., Knapstein P., Donat A., Winneberger J., Rosenthal L., Kohli P., Erdmann C., Fuchs M., Frosch K. H., Tsitsilonis S., Amling M., Schinke T. & Keller J. Procalcitonin is expressed in osteoblasts and limits bone resorption through inhibition of macrophage migration during intermittent PTH treatment. Bone Res 10, 9, doi:10.1038/s41413-021-00172-y (2022). 35087025 PMC8795393

[19] Muller B. & Becker K. L. Procalcitonin: how a hormone became a marker and mediator of sepsis. Swiss Med Wkly 131, 595–602, doi:10.4414/smw.2001.09751 (2001). 11820070

[20] Nagayama D., Imamura H., Endo K., Saiki A., Sato Y., Yamaguchi T., Watanabe Y., Ohira M., Shirai K. & Tatsuno I. Marker Of Sepsis Severity Is Associated With The Variation In Cardio-Ankle Vascular Index (CAVI) During Sepsis Treatment. Vasc Health Risk Manag 15, 509–516, doi:10.2147/VHRM.S228506 (2019). 31806982 PMC6842284

[21] Tang H., Huang T., Jing J., Shen H. & Cui W. Effect of procalcitonin-guided treatment in patients with infections: a systematic review and meta-analysis. Infection 37, 497–507, doi:10.1007/s15010-009-9034-2 (2009). 19826761

[22] Leli C., Ferranti M., Moretti A., Al Dhahab Z. S., Cenci E. & Mencacci A. Procalcitonin levels in gram-positive, gram-negative, and fungal bloodstream infections. Dis Markers 2015, 701480, doi:10.1155/2015/701480 (2015). 25852221 PMC4380090

[23] Sexton P. M., Christopoulos G., Christopoulos A., Nylen E. S., Snider R. H. Jr. & Becker K. L. Procalcitonin has bioactivity at calcitonin receptor family complexes: potential mediator implications in sepsis. Crit Care Med 36, 1637–1640, doi:10.1097/CCM.0b013e318170a554 (2008). 18434892

[24] Baranowsky A., Appelt J., Kleber C., Lange T., Ludewig P., Jahn D., Pandey P., Keller D., Rose T., Schetler D., Braumuller S., Huber-Lang M., Tsitsilonis S., Yorgan T., Frosch K. H., Amling M., Schinke T. & Keller J. Procalcitonin Exerts a Mediator Role in Septic Shock Through the Calcitonin Gene-Related Peptide Receptor. Crit Care Med 49, e41–e52, doi:10.1097/CCM.0000000000004731 (2021). 33196529

[25] Messerer D. A. C., Datzmann T., Baranowsky A., Peschel L., Hoffmann A., Groger M., Amling M., Wepler M., Nussbaum B. L., Jiang S., Knapstein P., Donat A., Calzia E., Radermacher P. & Keller J. Systemic calcitonin gene-related peptide receptor antagonism decreases survival in a porcine model of polymicrobial sepsis: blinded randomised controlled trial. Br J Anaesth 128, 864–873, doi:10.1016/j.bja.2021.11.042 (2022). 35131096

[26] Dobin A., Davis C. A., Schlesinger F., Drenkow J., Zaleski C., Jha S., Batut P., Chaisson M. & Gingeras T. R. STAR: ultrafast universal RNA-seq aligner. Bioinformatics 29, 15–21, doi:10.1093/bioinformatics/bts635 (2013). 23104886 PMC3530905

[27] Liao Y., Smyth G. K. & Shi W. featureCounts: an efficient general purpose program for assigning sequence reads to genomic features. Bioinformatics 30, 923–930, doi:10.1093/bioinformatics/btt656 (2014). 24227677

[28] Thorvaldsdottir H., Robinson J. T. & Mesirov J. P. Integrative Genomics Viewer (IGV): high-performance genomics data visualization and exploration. Brief Bioinform 14, 178–192, doi:10.1093/bib/bbs017 (2013). 22517427 PMC3603213

[29] Zheng G. X., Terry J. M., Belgrader P., Ryvkin P., Bent Z. W., Wilson R., Ziraldo S. B., Wheeler T. D., McDermott G. P., Zhu J., Gregory M. T., Shuga J., Montesclaros L., Underwood J. G., Masquelier D. A., Nishimura S. Y., Schnall-Levin M., Wyatt P. W., Hindson C. M., Bharadwaj R., Wong A., Ness K. D., Beppu L. W., Deeg H. J., McFarland C., Loeb K. R., Valente W. J., Ericson N. G., Stevens E. A., Radich J. P., Mikkelsen T. S., Hindson B. J. & Bielas J. H. Massively parallel digital transcriptional profiling of single cells. Nat Commun 8, 14049, doi:10.1038/ncomms14049 (2017). 28091601 PMC5241818

[30] Lewandowski B. C., Sukumaran S. K., Margolskee R. F. & Bachmanov A. A. Amiloride-Insensitive Salt Taste Is Mediated by Two Populations of Type III Taste Cells with Distinct Transduction Mechanisms. J Neurosci 36, 1942–1953, doi:10.1523/JNEUROSCI.2947-15.2016 (2016). 26865617 PMC4748077

[31] Sukumaran S. K., Lewandowski B. C., Qin Y., Kotha R., Bachmanov A. A. & Margolskee R. F. Whole transcriptome profiling of taste bud cells. Sci Rep 7, 7595, doi:10.1038/s41598-017-07746-z (2017). 28790351 PMC5548921

[32] Satija R., Farrell J. A., Gennert D., Schier A. F. & Regev A. Spatial reconstruction of single-cell gene expression data. Nat Biotechnol 33, 495–502, doi:10.1038/nbt.3192 (2015). 25867923 PMC4430369

[33] Qin Y., Palayyan S. R., Zheng X., Tian S., Margolskee R. F. & Sukumaran S. K. Type II taste cells participate in mucosal immune surveillance. PLoS Biol 21, e3001647, doi:10.1371/journal.pbio.3001647 (2023). 36634039 PMC9836272

[34] Bankhead P., Loughrey M. B., Fernandez J. A., Dombrowski Y., McArt D. G., Dunne P. D., McQuaid S., Gray R. T., Murray L. J., Coleman H. G., James J. A., Salto-Tellez M. & Hamilton P. W. QuPath: Open source software for digital pathology image analysis. Sci Rep 7, 16878, doi:10.1038/s41598-017-17204-5 (2017). 29203879 PMC5715110

[35] Qin Y., Sukumaran S. K., Jyotaki M., Redding K., Jiang P. & Margolskee R. F. Gli3 is a negative regulator of Tas1r3-expressing taste cells. PLoS Genet 14, e1007058, doi:10.1371/journal.pgen.1007058 (2018). 29415007 PMC5819828

[36] Sukumaran S. K., Yee K. K., Iwata S., Kotha R., Quezada-Calvillo R., Nichols B. L., Mohan S., Pinto B. M., Shigemura N., Ninomiya Y. & Margolskee R. F. Taste cell-expressed alpha-glucosidase enzymes contribute to gustatory responses to disaccharides. Proc Natl Acad Sci U S A 113, 6035–6040, doi:10.1073/pnas.1520843113 (2016). 27162343 PMC4889361

[37] Xie W., Fisher J. T., Lynch T. J., Luo M., Evans T. I., Neff T. L., Zhou W., Zhang Y., Ou Y., Bunnett N. W., Russo A. F., Goodheart M. J., Parekh K. R., Liu X. & Engelhardt J. F. CGRP induction in cystic fibrosis airways alters the submucosal gland progenitor cell niche in mice. J Clin Invest 125, 2179, doi:10.1172/JCI82138 (2015). PMC4463222 25932677

[38] Zhou Y., Feng Y., Liang X., Gui S., Ren D., Liu Y., She J., Zhang X., Song F., Yu L., Zhang Y., Wang J., Zou Z., Mei J., Wen S., Yang M., Li X., Tan X. & Li Y. Elevations in presepsin, PCT, hs-CRP, and IL-6 levels predict mortality among septic patients in ICU. J Leukoc Biol, doi:10.1093/jleuko/qiae121 (2024). 38776408

[39] Montavon P. & Lindstrand K. Immunohistochemical localization of neuron-specific enolase and calcitonin gene-related peptide in pig taste papillae. Regul Pept 36, 235–248, doi:10.1016/0167-0115(91)90059-p (1991). 1805299

[40] Montavon P. & Lindstrand K. Immunohistochemical localization of neuron-specific enolase and calcitonin gene-related peptide in rat taste papillae. Regul Pept 36, 219–233, doi:10.1016/0167-0115(91)90058-o (1991). 1805298

[41] Kusakabe T., Matsuda H., Gono Y., Furukawa M., Hiruma H., Kawakami T., Tsukuda M. & Takenaka T. Immunohistochemical localisation of regulatory neuropeptides in human circumvallate papillae. J Anat 192 (Pt 4), 557–564, doi:10.1046/j.1469-7580.1998.19240557.x (1998). 9723982 PMC1467809

[42] Park G. Y., Lee G., Yoon J., Han J., Choi P., Kim M., Lee S., Park C., Wu Z., Li Y. & Choi M. Glia-like taste cells mediate an intercellular mode of peripheral sweet adaptation. Cell 188, 141–156 e116, doi:10.1016/j.cell.2024.10.041 (2025). 39561773

[43] Michot B., Casey S. M. & Gibbs J. L. Effects of Calcitonin Gene-related Peptide on Dental Pulp Stem Cell Viability, Proliferation, and Differentiation. J Endod 46, 950–956, doi:10.1016/j.joen.2020.03.010 (2020). 32387076

[44] Zhao X., Wu G., Zhang J., Yu Z. & Wang J. Activation of CGRP receptor-mediated signaling promotes tendon-bone healing. Sci Adv 10, eadg7380, doi:10.1126/sciadv.adg7380 (2024). PMC10923525 38457499

[45] Kim Y. J. & Granstein R. D. Roles of calcitonin gene-related peptide in the skin, and other physiological and pathophysiological functions. Brain Behav Immun Health 18, 100361, doi:10.1016/j.bbih.2021.100361 (2021). 34746878 PMC8551410

[46] Li W., Zhang Z., Li X., Cai J., Li D., Du J., Zhang B., Xiang D., Li N. & Li Y. CGRP derived from cardiac fibroblasts is an endogenous suppressor of cardiac fibrosis. Cardiovasc Res 116, 1335–1348, doi:10.1093/cvr/cvz234 (2020). 31504241

[47] Wee N. K. Y., Novak S., Ghosh D., Root S. H., Dickerson I. M. & Kalajzic I. Inhibition of CGRP signaling impairs fracture healing in mice. J Orthop Res 41, 1228–1239, doi:10.1002/jor.25474 (2023). 36281531 PMC10123175

