# Supplementary material for: Expression of Calca gene-derived peptides in the murine taste system
Source: bioRxiv. 2026 Apr 1:2026.01.16.700005. Originally published 2026 Jan 20. Preprint. [Version 2] doi: 10.64898/2026.01.16.700005 (PMC12871746; doi:10.64898/2026.01.16.700005)
Supplement: Supplement 1 [file media-1.pdf]

Supplementary data

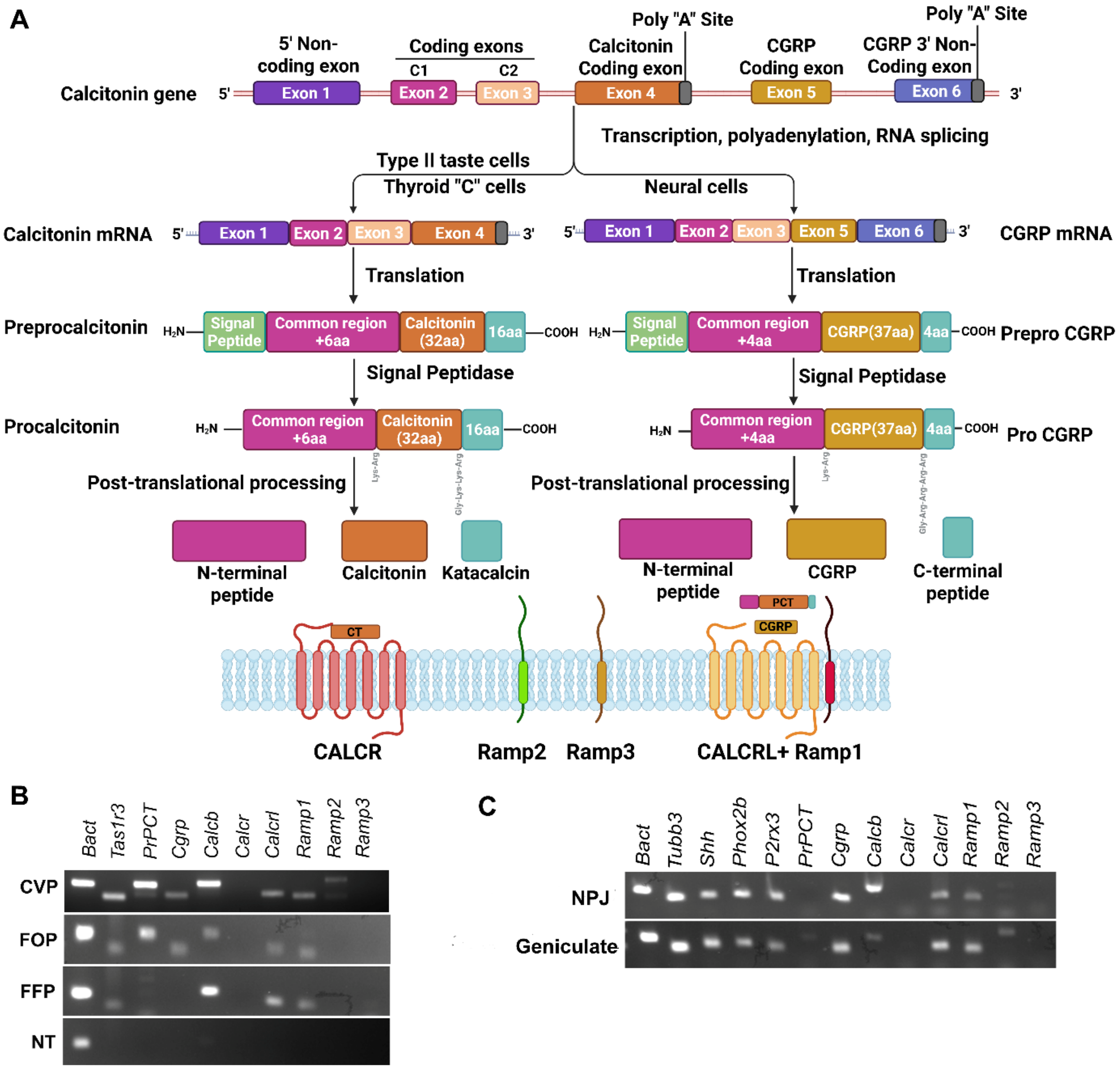

Figure S1. ***Calca*** **transcripts, peptides and their receptors, and their expression in taste papillae and ganglia**. **A)** The structure of the *Calca* gene, and the alternative splicing of the *Calca* mRNA that generates either the preprocalcitonin or pre proCGRP encoding transcripts. PCT, calcitonin (CT), Katacalcin and CGRP are produced by multistep proteolytic processing of the respective precursor proteins as shown. CT is a ligand for CALCR, while PCT and CGRP are ligands for CGRP1R, formed by a heterodimer of CALCRL and RAMP1. **B)** Expression of indicated transcripts in taste papillae and non-taste lingual epithelium (NT). *PrPCT, Calcb*, *Calcrl* and *Ramp1* expression is observed in CVP, FFP and

FOP. Weak *Cgrp expression* is observed in CVP and FOP, while it is undetectable in FOP and FFP. *Calcrl and Ramp1* are expressed in all taste papillae while *Calcr, Ramp2 and Ramp3 are* not detected in any tissue. None of the tested mRNAs are detected in NT. *Tas1r3* is used as a control to demonstrate the quality of taste cDNA, and *Bact* is used as cDNA synthesis control. **C)** qPCR of above transcripts in the geniculate and NPJ ganglia. *Cgrp*, *Calcb, Calcrl, Ramp1 and Ramp2* are expressed in both ganglia. Expression of *Ramp3*, *PrPCT* and *Calcr* is not observed in either ganglion. *Bact* is used as cDNA synthesis control, while the taste ganglion marker genes *Tubb3, Shh, Phox2b*, and *P2rx3* are used to demonstrate the quality of ganglia cDNA. Illustration in panel A was created in BioRender. Siddiqui, A. H. (2026) https://BioRender.com/gh3z1im

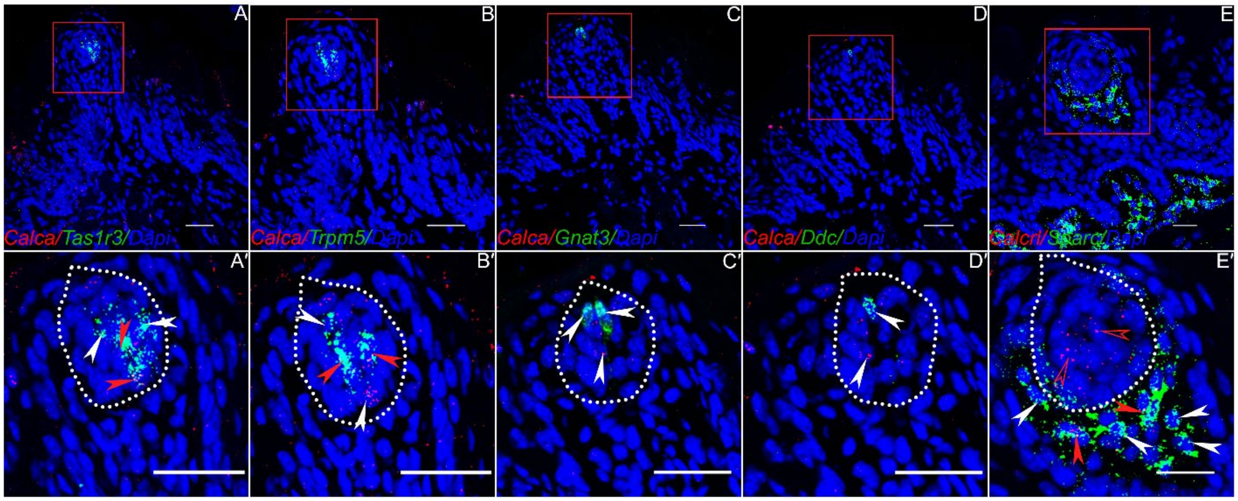

Figure S2. Expression of *Calca* and *Calcrl* in fungiform papillae. RNAscope Hiplex fluorescence assay was used to determine the coexpression of *Calca* with the taste cell markers *Tas1r3* (A-A'), *Trpm5* (B-B'), *Gnat3* (C-C'), *Ddc* (D-D') and of *Calcrl* with the fibroblast marker gene *Sparc*. The areas highlighted in red boxes in the top row are magnified in the bottom rows for each set. Taste buds are highlighted by white dotted lines. Areas inside white boxes in A-E are magnified in A'-E'. Weak expression of *Calca* is observed over all, and it appears to be coexpressed with *Tas1r3* and *Trpm5*. No coexpression is observed with *Gnat3* and *Ddc*. *Calcrl* expression is observed in basal (presumably stem/progenitor) cells in the taste buds and adjacent fibroblasts. Filled red arrowheads highlight double positive cells, and filled white arrowheads highlight single positive cells for taste/fibroblast marker genes, and open red arrowheads show single *Calcrl* positive cells. Scale bars = 30 µm. DAPI is used as a counterstain for nuclei.

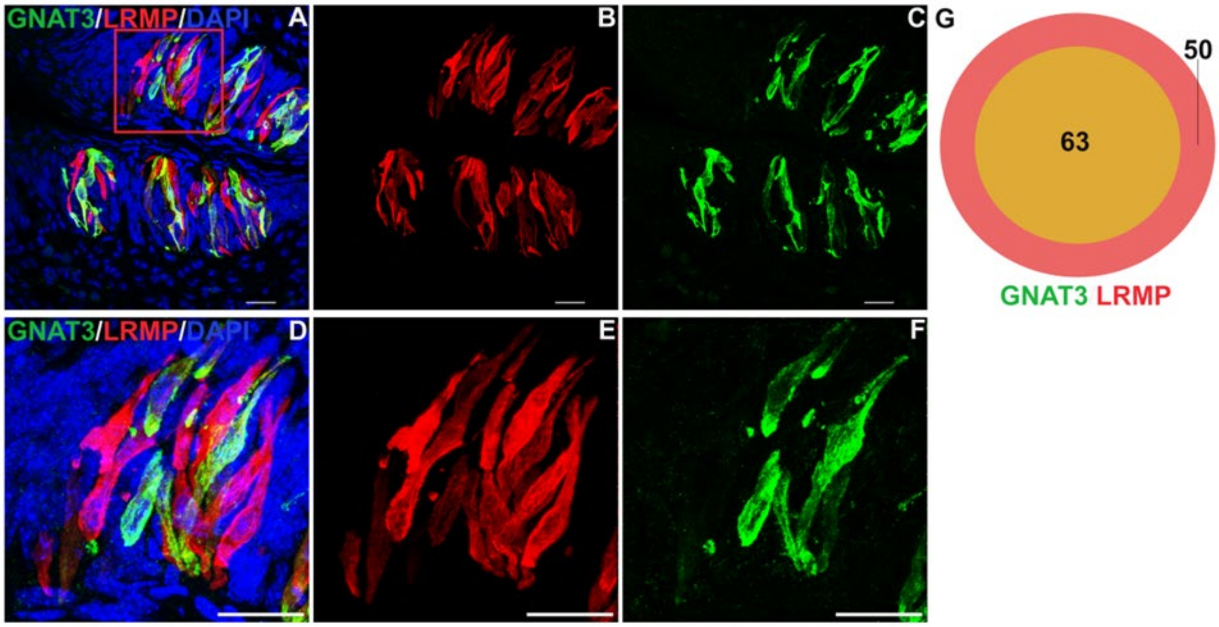

Figure S3. Double labeling for GNAT3 and LRMP. A-F) Unlike PCT staining shown in Figure 3, GNAT3 staining is restricted primarily to weaker LRMP expressing cells with less intense staining for LRMP. **G)** Venn diagram shows quantification of coexpression data. Scale bars= 30 µm. DAPI is used as a counterstain for nuclei.

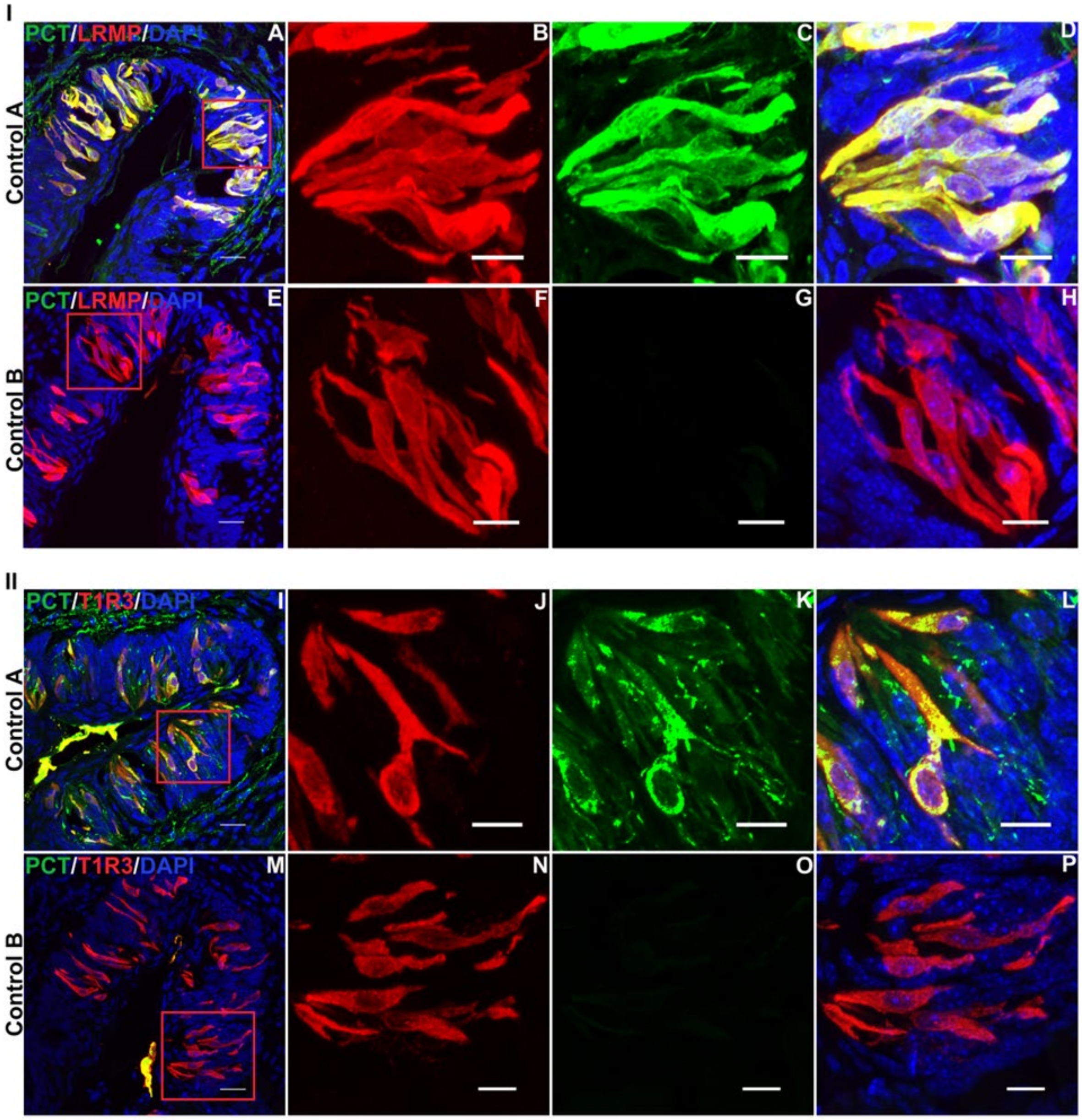

Figure S4. Controls for immunostaining with two rabbit primary antibodies. Double indirect immunofluorescence confocal microscopy of CVP shows cross-labeling when unlabelled donkey Fab fragment is omitted (control A, I & II), whereas adequate blocking of rabbit IgG is achieved with excess donkey Fab fragment (control B, I and II). Scalebars=30 μm. DAPI is used as a counterstain for nuclei.

Probe	CAT number	LOT number
RNAscope™ HiPlex Probe- Mm-Ddc-T1	318681-T1	22363A
RNAscope™ HiPlex Probe- Mm-Tas1r3-T2	515431-T2	22174B
RNAscope™ HiPlex Probe- Mm-Sparc-T4	466781-T4	24017A
RNAscope™ HiPlex Probe- Mm-Calcrl-T5	452281-T5	24017A
RNAscope™ HiPlex Probe- Mm-Calca-T6	578771-T6	223634
RNAscope™ HiPlex Probe- Mm-Gnat3-T8	531661-T8	22171A
RNAscope™ HiPlex Probe- Mm-Trpm5-T9	453251-T9	22363A
RNAscope™ HiPlex Probe- Mm-Arg1-T9	403441-T9	24017A
RNAscope™ HiPlex Probe- Mm-Ccr6-T11	424461-T11	24025A

Table S1: List of RNAscope HiPlex Probes used in this study.

Antibody	Host Species	Catalog No.	RRID	Source	Dilutions
PCT	Rabbit	LS-C296040	-	LS Bio, Newark, CA	1: 50
GNAT3	Goat	OAEB00418	AB_1088282	Aviva Systems Biology, San Diego, CA	1:500
LRMP	Rabbit	ORB166443	-	Biorbyt, Durham, NC	1:600
T1R3	Rabbit	-	-	Gift from Dr. Ichiro Matsumoto, Monell Chemical Senses center	1:500
CGRP	Goat	AB36001	AB_725807	Abcam Inc., Waltham, MA	1:250
TUBB3	Rabbit	AB18207	AB_444319	Abcam Inc., Waltham, MA	1:500

Alexa Fluor 488 Conjugated AffiniPure Fab Fragmant Goat anti-Rabbit IgG (H+L)	Goat	111-547-003	-	Jackson Immuno Research Inc. West Grove, PA	1:500
Alexa Fluor 647 Conjugated AffiniPure Fab Fragmant Donkey anti-Rabbit IgG (H+L)	Donkey	711-607-003	-	Jackson Immuno Research Inc. West Grove, PA	1:500
Alexa Fluor 647 Conjugated AffiniPure Donkey anti-Goat IgG (H+L)	Donkey	705-606-147	-	Jackson Immuno Research Inc. West Grove, PA	1:500
Alexa Fluor 488 Conjugated Donkey anti-Goat IgG	Donkey	A11055	-	Jackson Immuno Research Inc. West Grove, PA	1:500
AffiniPure Fab Fragment Donkey anti-Rabbit IgG(H+L)	Donkey	711-007-003	-	Jackson Immuno Research Inc. West Grove, PA	1:500

Table S2: Primary and secondary antibodies for IHC and western blot used in this study.

Gene	Forward Primer	Reverse Primer
Bact	GGCTGTATTCCCCTCCACG	CCAGTTGGTAACAATGCCATGT
Calcrl	CATCGTGGTGGCTGTGTTT	GTAATACAAGCTTCTGGCAATGG
Calcr	GCTGAGTGCAGAAACCCACT	TTTGCCTCATCTTGGTCACA
Ramp1	AGCCGCTTCAAGGAGAACAT	CGTGCTTGGTGCAGTAAGTG
Ramp2	TGAGGACAGCCTTGTGTCAA	CAGCACAGCAGAAAGGTTCC
Ramp3	AAGTTGGTTTTGGACGGTGA	GCATACCTGGGCACACTCA
Tubb3	GAACCTGGAACCATGGACAG	GTTGTTGCCAGCACCACTCT
Shh	AAGGATGAGGAAAACACGGG	GGTCACTCGCAGCTTCACTC
Phox2b	CACTTTTGGGGCCACGTC	CGTGGTCGGTGAAGAGTTTG
P2rx3	CCAAATATTCCTTCACTCGGC	GCTGCCATTCTCCATCTTGT
PrePCT	GGAGCAGGAGGAAGAGCAGG	GCCAGGTGCTTCAACCCCAA
Cgrp	TATGCAGATGAAAGCCAGGG	GTGGCAGTGTTGCAGGATCT
Calcb	CAGGAAGCTGGAACAGGAGG	AAGGCTTCAGAGCCCACATC
Tas1r3	CAAGTTCTTCAGCTTCTTCC	GGCGGCCACCCAGTTCCAGC

Table S3: List of primers used for PCR and qPCR.
