# Supplementary material for: Expression of Calca gene-derived peptides in the murine taste system
Source: bioRxiv. 2026 Jan 20:2026.01.16.700005. Preprint. [Version 1] doi: 10.64898/2026.01.16.700005 (PMC12871746; doi:10.64898/2026.01.16.700005)
Supplement: Supplementary file 1 [file NIHPP2026.01.16.700005V1-supplement-1.pdf]

bioRxiv preprint doi: https://doi.org/10.64898/2026.01.16.700005; this version posted January 20, 2026. The copyright holder has placed this preprint (which was not certified by peer review) in the Public Domain. It is no longer restricted by copyright. Anyone can legally share, reuse, remix, or adapt this material for any purpose without crediting the original authors.

Supplementary data

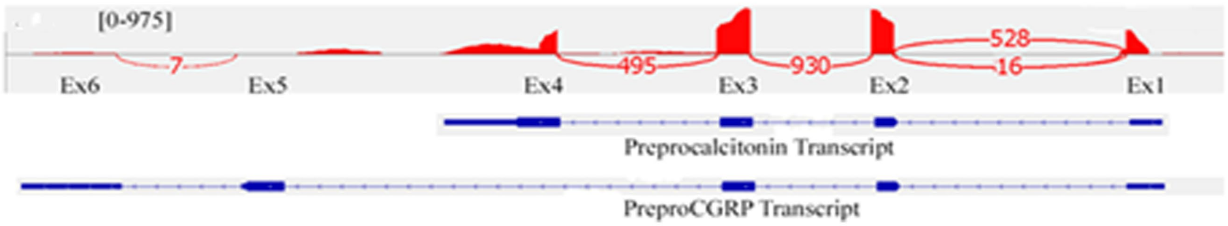

Figure S1. Sashimi plot of the *Calca* gene locus (transcribed from bottom strand in right to left direction) from bulk RNASeq data from CVP. The reads aligning to exons 1-6 (Ex1-Ex6, right to left) are shown in red, with the height of the red bars showing the strength of expression, and the number of reads aligning across splice junctions indicated in the loops between exons. No reads align to the preproCGRP specific exon 5, while large number of reads align to prePCT specific exon 4.

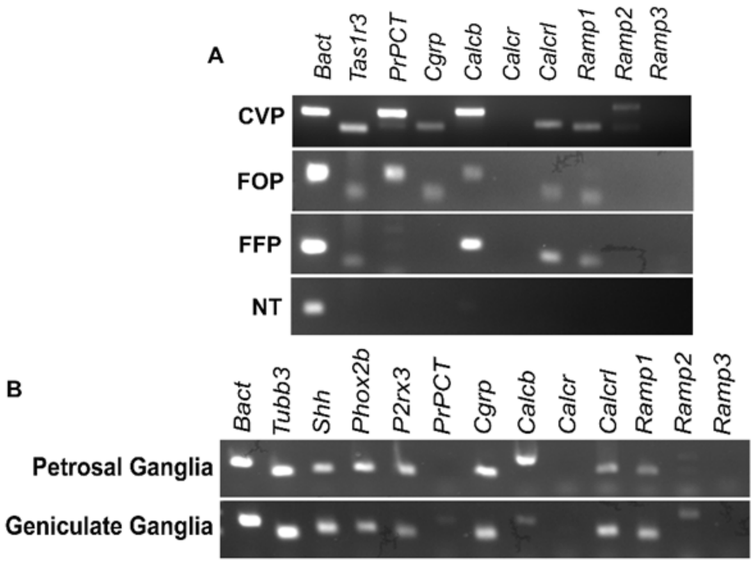

Figure S2. End point PCR showing expression of Calca- related mRNAs. **A)** Expression of indicated transcripts in taste papillae and non-taste lingual epithelium (NT). *PrPCT, Calcb*, *Calcrl* and *Ramp1* expression is observed in CVP, FFP and FOP. Weak *Cgrp expression* is observed in CVP and FOP, while it is undetectable in FOP and FFP. *Calcrl and Ramp1* are expressed in all taste papillae while *Calcr, Ramp2 and Ramp3 are* not detected in any tissue. None of the tested mRNAs are detected in NT. *Tas1r3* is used as a control to demonstrate the quality of taste cDNA, and *Bact* is used as cDNA synthesis control. **B)** qPCR of above transcripts in the geniculate and nodose-petrosal ganglia. *Cgrp*, *Calcb, Calcrl, Ramp1 and Ramp2* are expressed in both ganglia. Expression of *Ramp3*, *PrPCT* and *Calcr* is not observed in either ganglion. *Bact* is used as cDNA synthesis control, while the taste ganglion marker genes *Tubb3, Shh, Phox2b*, and *P2rx3* are used to demonstrate the quality of ganglia cDNA.

bioRxiv preprint doi: https://doi.org/10.64898/2026.01.16.700005; this version posted January 20, 2026. The copyright holder has placed this preprint (which was not certified by peer review) in the Public Domain. It is no longer restricted by copyright. Anyone can legally share, reuse, remix, or adapt this material for any purpose without crediting the original authors.

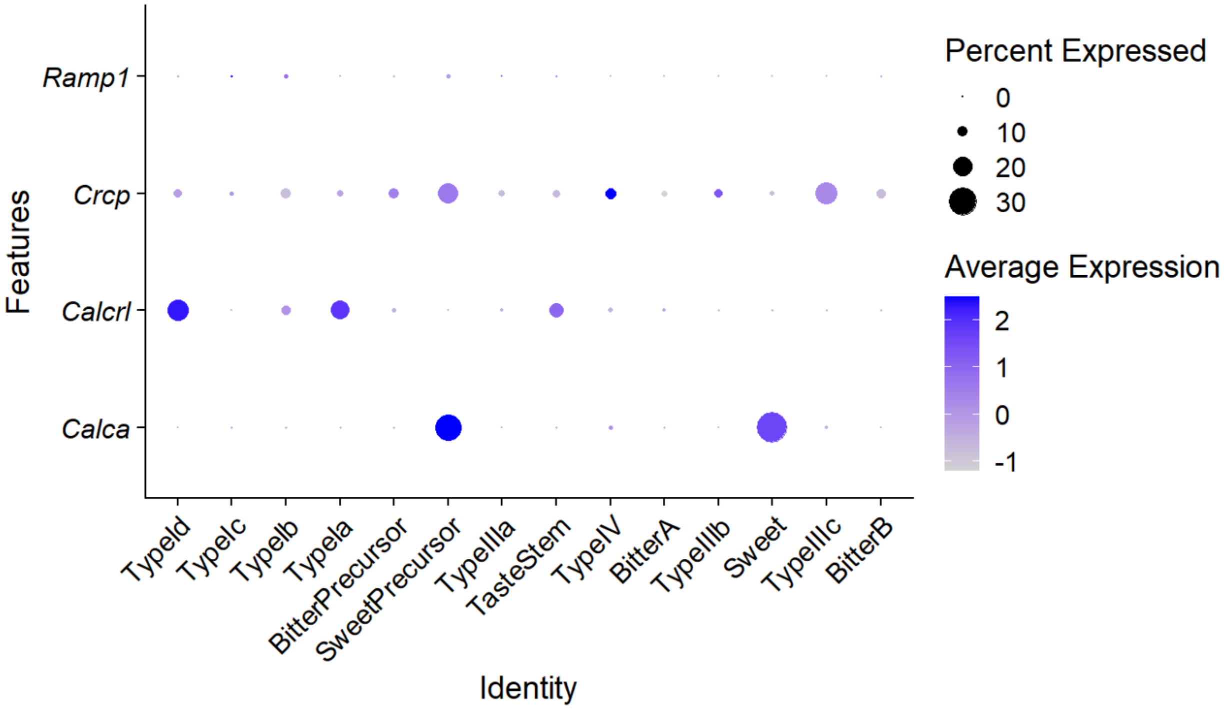

Figure S3. Dot plot from CVP. Type I a-d, type IIIa-c and type IV, sweet and bitter taste receptor cells are detected. BitterPrecursor and sweetPrecursor are clusters of Immature type II immature cells. TasteStem is a cluster of stem cells. Strong *Calca* (=prePCT) mRNA expression is seen in sweet taste receptor cells and immature type II cells, and *Calcrl* expression is observed in type I and taste stem cells, and *Crcp* expression is found in multiple cell types. *Ramp1* could not be detected in this dataset.

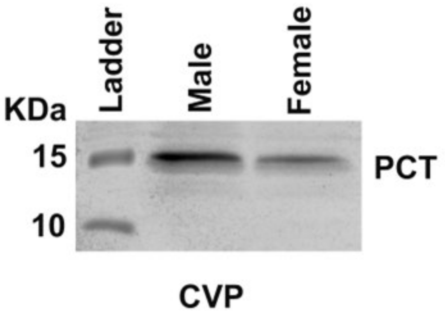

Figure S4. Western blot analysis of protein extract from CVP of male and female mice with anti-PCT antibody showing expression of PCT.

bioRxiv preprint doi: https://doi.org/10.64898/2026.01.16.700005; this version posted January 20, 2026. The copyright holder has placed this preprint (which was not certified by peer review) in the Public Domain. It is no longer restricted by copyright. Anyone can legally share, reuse, remix, or adapt this material for any purpose without crediting the original authors.

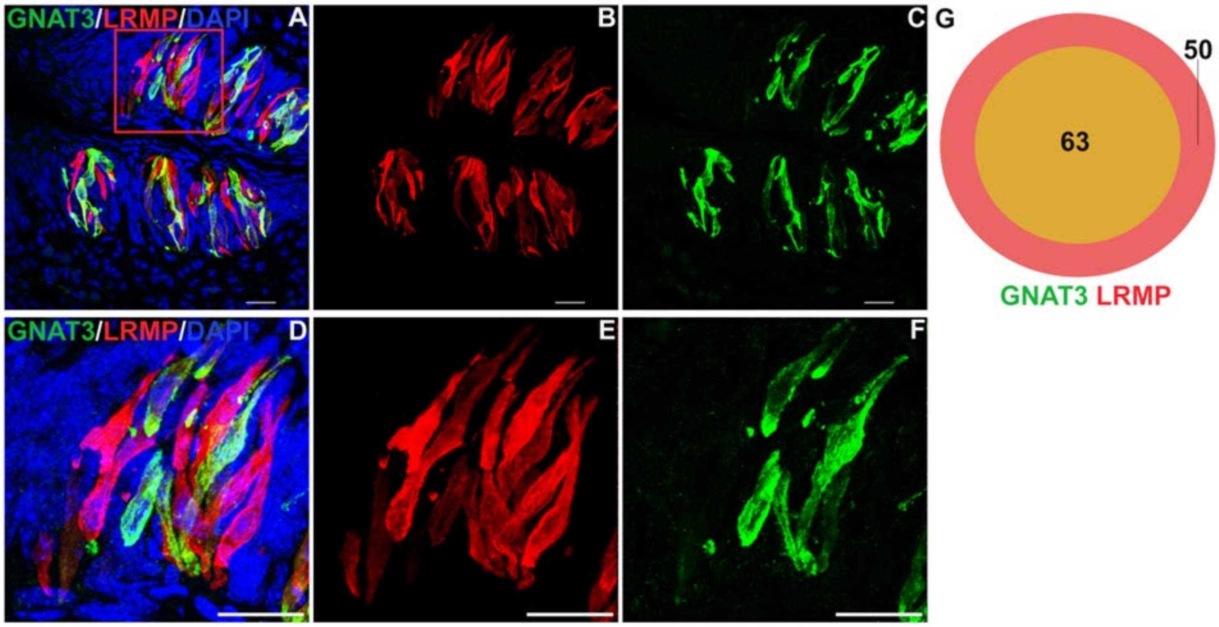

Figure S5. Double labeling for GNAT3 and LRMP. A-F) Unlike PCT staining shown in Figure 3, GNAT3 staining is restricted to weaker LRMP expressing cells with less intense staining for LRMP. **G)** Venn diagram shows quantification of coexpression data.

bioRxiv preprint doi: https://doi.org/10.64898/2026.01.16.700005; this version posted January 20, 2026. The copyright holder has placed this preprint (which was not certified by peer review) in the Public Domain. It is no longer restricted by copyright. Anyone can legally share, reuse, remix, or adapt this material for any purpose without crediting the original authors.

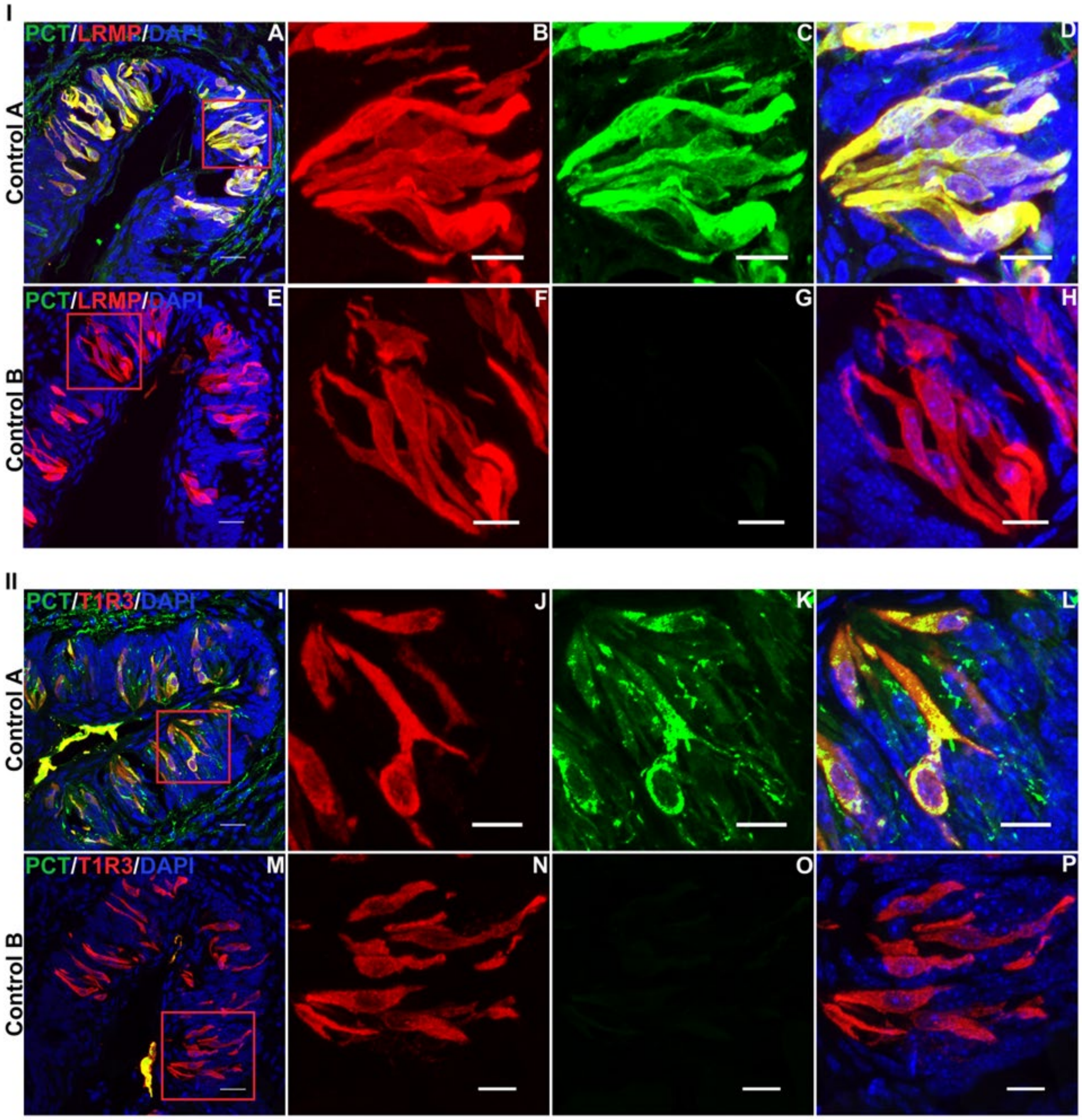

Figure S6. Controls for immunostaining with two rabbit primary antibodies. Double indirect immunofluorescence confocal microscopy of CVP shows cross-labeling when unlabelled donkey Fab fragment is omitted (control A, *I* & *II*), whereas adequate blocking of rabbit IgG is achieved with excess donkey Fab fragment (control B, I and II).

bioRxiv preprint doi: https://doi.org/10.64898/2026.01.16.700005; this version posted January 20, 2026. The copyright holder has placed this preprint (which was not certified by peer review) in the Public Domain. It is no longer restricted by copyright. Anyone can legally share, reuse, remix, or adapt this material for any purpose without crediting the original authors.

Probe	CAT number	LOT number
RNAscope™ HiPlex Probe- Mm-Ddc-T1	318681-T1	22363A
RNAscope™ HiPlex Probe- Mm-Tas1r3-T2	515431-T2	22174B
RNAscope™ HiPlex Probe- Mm-Sparc-T4	466781-T4	24017A
RNAscope™ HiPlex Probe- Mm-Calcrl-T5	452281-T5	24017A
RNAscope™ HiPlex Probe- Mm-Calca-T6	578771-T6	223634
RNAscope™ HiPlex Probe- Mm-Gnat3-T8	531661-T8	22171A
RNAscope™ HiPlex Probe- Mm-Trpm5-T9	453251-T9	22363A

Table S1: List of RNAscope HiPlex Probes used in this study.

Antibody	Host Species	Catalog No.	RRID	Source	Dilutions
PCT	Rabbit	LS-C296040	-	LS Bio, Newark, CA	1: 50
GNAT3	Goat	OAEB00418	AB_1088282	Aviva Systems Biology, San Diego, CA	1:500
LRMP	Rabbit	ORB166443	-	Biorbyt, Durham, NC	1:600
T1R3	Rabbit	-	-	Gift from Dr. Ichiro Matsumoto, Monell Chemical Senses center	1:500
Alexa Fluor 488 Conjugated AffiniPure Fab Fragmant Goat anti-Rabbit IgG (H+L)	Goat	111-547-003	-	Jackson Immuno Research Inc. West Grove, PA	1:500

bioRxiv preprint doi: https://doi.org/10.64898/2026.01.16.700005; this version posted January 20, 2026. The copyright holder has placed this preprint (which was not certified by peer review) in the Public Domain. It is no longer restricted by copyright. Anyone can legally share, reuse, remix, or adapt this material for any purpose without crediting the original authors.

Alexa Fluor 647 Conjugated AffiniPure Fab Fragmant Donkey anti-Rabbit IgG (H+L)	Donkey	711-607-003	-	Jackson Immuno Research Inc. West Grove, PA	1:500
Alexa Fluor 647 Conjugated AffiniPure Donkey anti-Goat IgG (H+L)	Donkey	705-606-147	-	Jackson Immuno Research Inc. West Grove, PA	1:500
Alexa Fluor 488 Conjugated Donkey anti-Goat IgG	Donkey	A11055	-	Jackson Immuno Research Inc. West Grove, PA	1:500
AffiniPure Fab Fragment Donkey anti-Rabbit IgG(H+L)	Donkey	711-007-003	-	Jackson Immuno Research Inc. West Grove, PA	1:500

Table S2: Primary and secondary antibodies for IHC and western blot used in this study.

Gene	Forward Primer	Reverse Primer
Bact	GGCTGTATTCCCCTCCACG	CCAGTTGGTAACAATGCCATGT
Calcrl	CATCGTGGTGGCTGTGTTT	GTAATACAAGCTTCTGGCAATGG
Calcr	GCTGAGTGCAGAAACCCACT	TTTGCCTCATCTTGGTCACA
Ramp1	AGCCGCTTCAAGGAGAACAT	CGTGCTTGGTGCAGTAAGTG
Ramp2	TGAGGACAGCCTTGTGTCAA	CAGCACAGCAGAAAGGTTCC
Ramp3	AAGTTGGTTTTGGACGGTGA	GCATACCTGGGCACACTCA
Tubb3	GAACCTGGAACCATGGACAG	GTTGTTGCCAGCACCACTCT

bioRxiv preprint doi: https://doi.org/10.64898/2026.01.16.700005; this version posted January 20, 2026. The copyright holder has placed this preprint (which was not certified by peer review) in the Public Domain. It is no longer restricted by copyright. Anyone can legally share, reuse, remix, or adapt this material for any purpose without crediting the original authors.

Shh	AAGGATGAGGAAAACACGGG	GGTCACTCGCAGCTTCACTC
Phox2b	CACTTTTGGGGCCACGTC	CGTGGTCGGTGAAGAGTTTG
P2rx3	CCAAATATTCCTTCACTCGGC	GCTGCCATTCTCCATCTTGT
PrePCT	GGAGCAGGAGGAAGAGCAGG	GCCAGGTGCTTCAACCCCAA
Cgrp	TATGCAGATGAAAGCCAGGG	GTGGCAGTGTTGCAGGATCT
Calcb	CAGGAAGCTGGAACAGGAGG	AAGGCTTCAGAGCCCACATC
Tas1r3	CAAGTTCTTCAGCTTCTTCC	GGCGGCCACCCAGTTCCAGC

Table S3: List of primers used for PCR and qPCR.
